# An αB-Crystallin Peptide Rescues Compartmentalization and Trafficking Response to Cu Overload of ATP7B-H1069Q, the Most Frequent Cause of Wilson Disease in the Caucasian Population

**DOI:** 10.3390/ijms19071892

**Published:** 2018-06-27

**Authors:** Simona Allocca, Michela Ciano, Maria Camilla Ciardulli, Chiara D’Ambrosio, Andrea Scaloni, Daniela Sarnataro, Maria Gabriella Caporaso, Massimo D’Agostino, Stefano Bonatti

**Affiliations:** 1 Department of Molecular Medicine and Medical Biotechnology, University of Naples Federico II, via S. Pansini 5, 80131 Naples, Italy; simal89@libero.it (S.A.); michelaciano@libero.it (M.C.); marikaciardulli93@libero.it (M.C.C.); sarnatar@unina.it (D.S.); mariagabriella.caporaso@unina.it (M.G.C.); massimo.dagostino@unina.it (M.D.); 2 Proteomics & Mass Spectrometry Laboratory, ISPAAM, National Research Council, Via Argine 1085, 80147 Naples, Italy; chiara.dambrosio@cnr.it (C.D.); andrea.scaloni@ispaam.cnr.it (A.S.); 3 Ceinge Biotecnologie avanzate scarl, via G. Salvatore 486, 80145 Naples, Italy

**Keywords:** ATP7B, Wilson disease, αB-crystallin, pharmacological peptide

## Abstract

The H1069Q substitution is the most frequent mutation of the Cu transporter ATP7B that causes Wilson disease in the Caucasian population. ATP7B localizes to the Golgi complex in hepatocytes, but, in the presence of excessive Cu, it relocates to the endo-lysosomal compartment to excrete Cu via bile canaliculi. In contrast, ATP7B-H1069Q is strongly retained in the ER, does not reach the Golgi complex and fails to move to the endo-lysosomal compartment in the presence of excessive Cu, thus causing toxic Cu accumulation. We have previously shown that, in transfected cells, the small heat-shock protein αB-crystallin is able to correct the mislocalization of ATP7B-H1069Q and its trafficking in the presence of Cu overload. Here, we first show that the α-crystallin domain of αB-crystallin mimics the effect of the full-length protein, whereas the N- and C-terminal domains have no such effect. Next, and most importantly, we demonstrate that a twenty-residue peptide derived from the α-crystallin domain of αB-crystallin fully rescues Golgi localization and the trafficking response of ATP7B-H1069Q in the presence of Cu overload. In addition, we show that this peptide interacts with the mutant transporter in the live cell. These results open the way to attempt developing a pharmacologically active peptide to specifically contrast the Wilson disease form caused by the ATP7B-H1069Q mutant.

## 1. Introduction

Wilson disease (WD) is a relatively frequent inherited liver disorder (about 1:7000 birth cases) characterized by a slow onset of insurgence [1,2]. WD is due to mutation in the Cu transporter protein ATP7B, an efflux transporter whose malfunction determines toxic accumulation of Cu in the liver with severe symptoms that may be followed by neurological problems [3,4,5]. Intriguingly, many mutations have been reported to alter the function of ATP7B, but over 50% of the patients in the Caucasian population carry the H1069Q substitution, either in homozygosity or compound heterozygosity [6]. Overexpression studies of ATP7B-H1069Q in different cell lines have allowed for elucidating the molecular defects of this mutated transporter [7,8,9,10,11]. ATP7B is localized in the trans-Golgi network (TGN) of the hepatocytes and pumps Cu from the cytosol into the lumen where Cu ions are captured by protein acceptors such as the serum protein Ceruloplasmin [12,13]. However, in the presence of Cu excess, ATP7B leaves the Golgi and reaches the endo-lysosomal compartment and the plasma membrane of bile canaliculi to excrete Cu in the bile [14]. In contrast, ATP7B-H1069Q cannot reach the Golgi complex due to being partially misfolded and thus remains in the endoplasmic reticulum (ER). Most importantly, in the presence of excess Cu, this mutant does not move to the endo-lysosomal compartment and therefore does not provide Cu excretion. Intriguingly, although the H1069Q substitution resides in and influences the nucleotide binding domain of the transporter, quite a significant residual level of transport activity is still borne by the mutant protein [9,15]. Unfortunately, this residual transport activity is useless (and probably deleterious), while it would be most likely sufficient to prevent/largely ameliorate the disease if the protein would be first correctly located in the TGN and then able to reach the endo-lysosomal compartment in the presence of Cu overload [16]. The current therapies for WD target either Cu adsorption in the intestine or favor its urinary excretion [3,4,5]. Albeit helpful, these treatments cause significant side effects and are of limited efficacy in several cases [17,18]. Thus, alternative and more specific therapeutic approaches are needed.

We have previously shown that the small heath shock protein αB-crystallin (HspB5 or CRYAB) is specifically able to prevent aggregation of ATP7B-H1069Q and efficiently restore both Golgi localization and trafficking to the endo-lysosomal compartment of the mutant transporter [10]. It is well known that CRYAB binds to proteins and prevents their aggregation through an ATP-independent holdase activity [19,20], forming large and dynamic homo- and hetero-oligomeric complexes that are thought to be instrumental in carrying out the holdase activity [19]. However, the holdase activity is maintained in vitro when peptides from the conserved α-crystallin domain of CRYAB are used in anti-aggregation tests [21]. Most surprisingly, the DRFSVNLDVKHFSPEELKVK peptide, corresponding to residues 73–92 of the α-Crystallin domain of CRYAB (henceforth referred to as Pept 73–92), has anti-apoptotic properties in human lens epithelial cells, in mouse organ-cultured lenses and in human fetal Retinal Pigment Endothelial cells [22,23]. In addition, this peptide has shown therapeutic effects in experimental autoimmune encephalomyelitis in mice and in cataract development in selenite-treated rats [22,24]. Despite the fact that, for all' aforementioned cases, the precise target(s) and the mechanism of action of Pept 73–92 have yet to be determined, these findings strongly suggest the possibility that the peptide could be used as a pharmacological chaperone [25]. Therefore, in order to develop a new and specific treatment for the WD form caused by the mutant ATP7B-H1069Q, we tested whether smaller segments of CRYAB protein could have the same rescue effect on this mutant borne by the full-length protein. Here, we report the striking effects obtained with the Pept 73–92 on the localization and trafficking of ATP7B-H1069Q. Furthermore, we demonstrate also its interaction with the mutant transporter in the live cell.

## 2. Results

### 2.1. The α-Crystallin Domain of CRYAB Rescues Golgi Localization of the ATP7B-H1069Q Mutant

**Figure 1:**
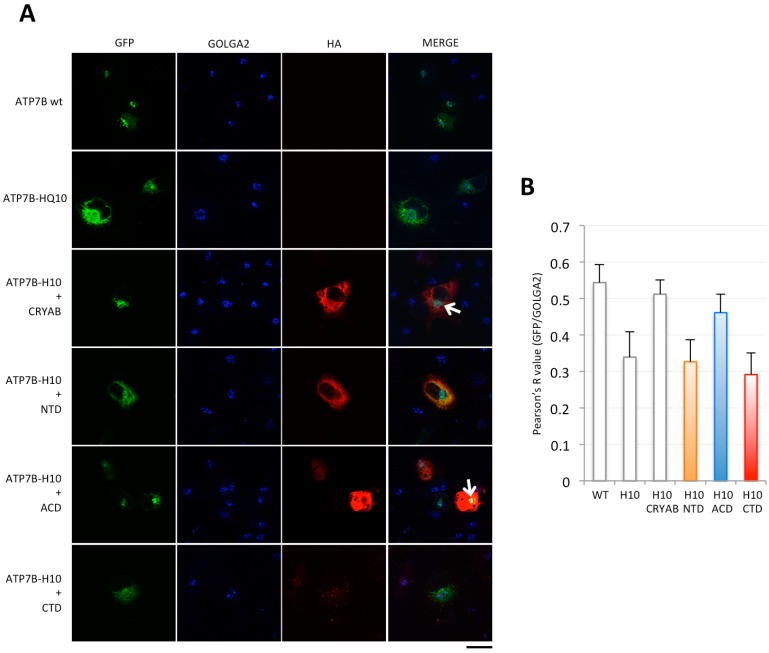
The α-Crystallin domain of CRYAB rescues Golgi localization of ATP7B-H1069Q mutant. (**A**) immunofluorescence analysis of COS7 cells transfected to express GFP-tagged ATP7B or ATP7B-H1069Q (ATP7B-H10) alone, or co-transfected to express full-length HA-tagged CRYAB or the isolated HA-tagged N-terminal (NTD), α-crystallin (ACD) and C-terminal (CTD) domains, as indicated on the left. After fixation, the cells were processed for triple confocal immunofluorescence analysis to reveal the Golgi complex with the marker GOLGA2. Note that the CTD was expressed at much lower level. White arrows point to region of GFP and GOLGA2 overlapping signal. Scale bar: 10 μm; (**B**) colocalization analysis of GFP and GOLGA2 signals (mean ± SD, *n* = 20 cells from two independent experiments). Abbreviations as in A.

First, we asked whether any of the isolated portions of CRYAB could maintain the ability of the full-length protein to rescue the Golgi localization of ATP7B-H1069Q in transfected cells. Indeed, partial activity of the truncated portion of CRYAB was previously observed in chaperone assays performed in vitro to counteract insulin aggregation [26]. Thus, we dissected CRYAB in its three main domains (Figure S1). In particular, the N-terminal, α-crystallin and C-terminal domains (corresponding to residues 1–66, 65–146 and 148–175, respectively) were cloned to generate HA-tagged constructs. Then, COS7 cells were transiently transfected to express Green fluorescent protein (GFP)-tagged ATP7B or ATP7B-H1069Q, or co-transfected with Hemagglutinin (HA)-CRYAB or the HA-tagged CRYAB constructs. Triple confocal immunofluorescence analysis revealed that, when ATP7B-H1069Q is co-transfected with the N- or C-terminal domains (marked as NTD and CTD, respectively), no rescue effect was obtained as the mutated protein is still retained in the ER (Figure 1A). In contrast, the α-crystallin domain (ACD) markedly rescues the proper intracellular compartmentalization of ATP7B-H1069Q, as evidenced by the clear localization in the Golgi complex of the mutated transporter (Figure 1A), to an extent almost identical to ATP7B or ATP7B-H1069Q rescued by full-length CRYAB. To quantify this observation, the co-localization of GFP and of the Golgi marker protein GOLGA2 signals was evaluated with the ImageJ Fiji 2.0.0 (National Institutes of Health, Bethesda, Maryland USA) co-localization plugin. The calculated Pearson's R values fully confirmed the obtained conclusion (Figure 1B).

### 2.2. Internalization and Distribution of Pept 73–92 in COS7 Cells

The results illustrated above prompted us to focus on the α-Crystallin domain of CRYAB to find the smallest possible portion of the protein able to rescue the Golgi-localization of ATP7B-H1069Q. Thus, in line with the previous reports showing the activity in vitro and in vivo of Pept 73–92 derived from the α-Crystallin domain [21,22,23,24], we analyzed the effect of this synthetic peptide, as opposed to control peptides (Figure S1). Initial experiments were performed with the tetramethylrhodamine azide (TAMRA) tagged version of the peptides (Figure S2), in order to allow their visualization. COS7 cells were incubated from 1 up to 20 h with 12.5 μM of each peptide and then processed for confocal immunofluorescence analysis. We observed that the fluorescent signal of the peptides was already detectable inside the cells after 1 h and partially co-localized with the lysosomal marker LAMP-1 after 20 h (Figure S2). It is worth noting that we have used the PULSin reagent (Polyplus-transfection Inc., New York, NY, USA) to deliver the TAMRA-tagged peptide in the cells, but the uptake of the peptide resulted only moderately increased, thus showing a good entry capacity of the peptide in COS7 cells. Next, we cell fractionated by differential centrifugation homogenates obtained from cells incubated as above (as schematically depicted in Figure S3A), and determined the amount of fluorescent peptide contained in the fractions by spectrofluorimetric analyses. As shown in Figure S3B, we found more than 60% of the peptides in the soluble S100 fraction and the remaining portion in the membrane-associated P16 and P100 fractions, indicating that large portions of the exogenously added peptides were indeed available in the cytosol of the cells. In both assays, no differences were detected between Pept 73–92 and its control peptide (Figures S2 and S3B). Finally, cell viability was tested with 3-(4,5-Dimethylthiazol-2-yl)-2,5-Diphenyltetrazolium Bromide (MTT) assays. As shown in Figure S3C, no toxic effect of the peptides on COS7 cells was observed.

### 2.3. Pept 73–92 Specifically Rescues Golgi Localization of ATB7B-H1069Q

**Figure 2:**
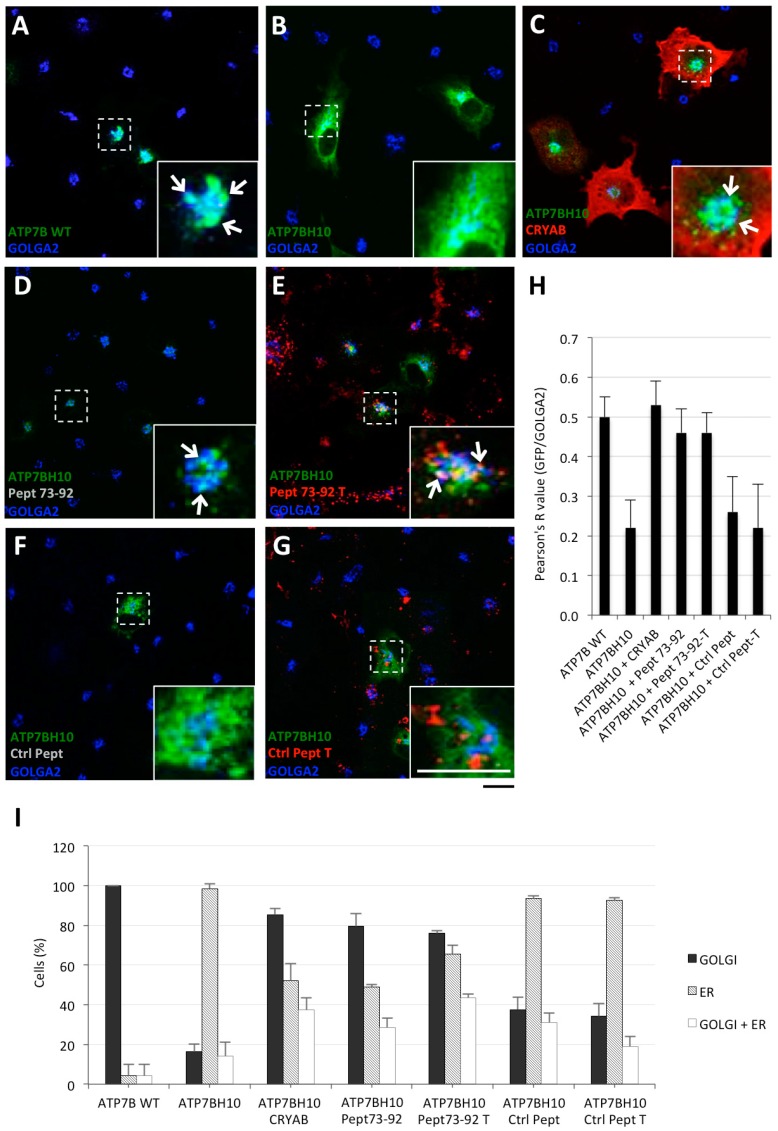
Pept 73–92 specifically rescues Golgi localization of ATB7B-H1069Q. (**A**–**G**) immunofluorescence analysis of COS7 cells transfected to express GFP-tagged ATP7B or ATP7B-H1069Q alone, or co-transfected to express full-length HA-tagged CRYAB or incubated with TAMRA-tagged (T) or untagged Pept 73–92 or control peptide 1 (Ctrl Pept), as indicated in each panel. After fixation, the cells were processed for triple confocal immunofluorescence analysis to reveal the Golgi complex with the marker GOLGA2. White arrows as in Figure 1. Scale bar: 10 μm; (**H**) colocalization analysis of GFP and GOLGA2 signals (means ± SD, *n* = 15 cells from three independent experiments). (**I**) Quantification of the percentage of cells exhibiting exhibiting ER, Golgi complex, and both ER and Golgi complex GFP staining (means ± SD, *n* = 25 cells from two independent experiments). Abbreviations as in Figure 1.

To assess whether Pept 73–92 was able to rescue the proper localization of ATP7B-H1069Q, as already established for the α-crystallin domain and full-length CRYAB, COS7 cells were either transfected to express GFP-tagged ATP7B or ATP7B-H1069Q, or co-transfected with full-length CRYAB as control. Next, the cells were incubated with either Pept 73–92 or with control peptides, tagged or untagged with TAMRA, from 24 to 48 h post transfection. Cells were then fixed and processed for confocal immunofluorescence analysis. As expected, ATP7B mainly localized in the Golgi complex, as indicated by co-localization with GOLGA2 protein (blue staining), whereas ATP7B-H1069Q was mainly present in the ER (Figure 2A,B). Most interestingly, in cells expressing ATP7B-H1069Q and either co-transfected with full-length CRYAB or incubated with untagged or TAMRA-tagged Pept 73–92, ATP7B-H1069Q showed Golgi localization as well as ATP7B (Figure 2C–E). In contrast, no such effect was observed in cells incubated with any version of the control peptide (Figure 2F,G).

To quantify these data, the extent of co-localization of GFP and GOLGA2 signals was measured with the ImageJ co-localization plugin and the obtained Pearson's R values are shown in Figure 2H. In addition, the percentage of cells exhibiting ER, Golgi complex, and ER + Golgi complex GFP staining was measured (Figure 2I). All together, these quantified results show that, in response to Pept 73–92 treatment (Figure 2D,E), ATP7B-H1069Q reaches the Golgi localization, thanks to Pept 73–92, almost as effectively as ATP7B alone or full-length CRYAB-corrected ATP7B-H1069Q (as reported in Figure 2A,C, respectively).

### 2.4. ATP7B-H1069Q Localized in the Golgi Complex Thanks to Pept 73–92 Moves to Post-Golgi Vesicles in Response to Cu Overload

**Figure 3:**
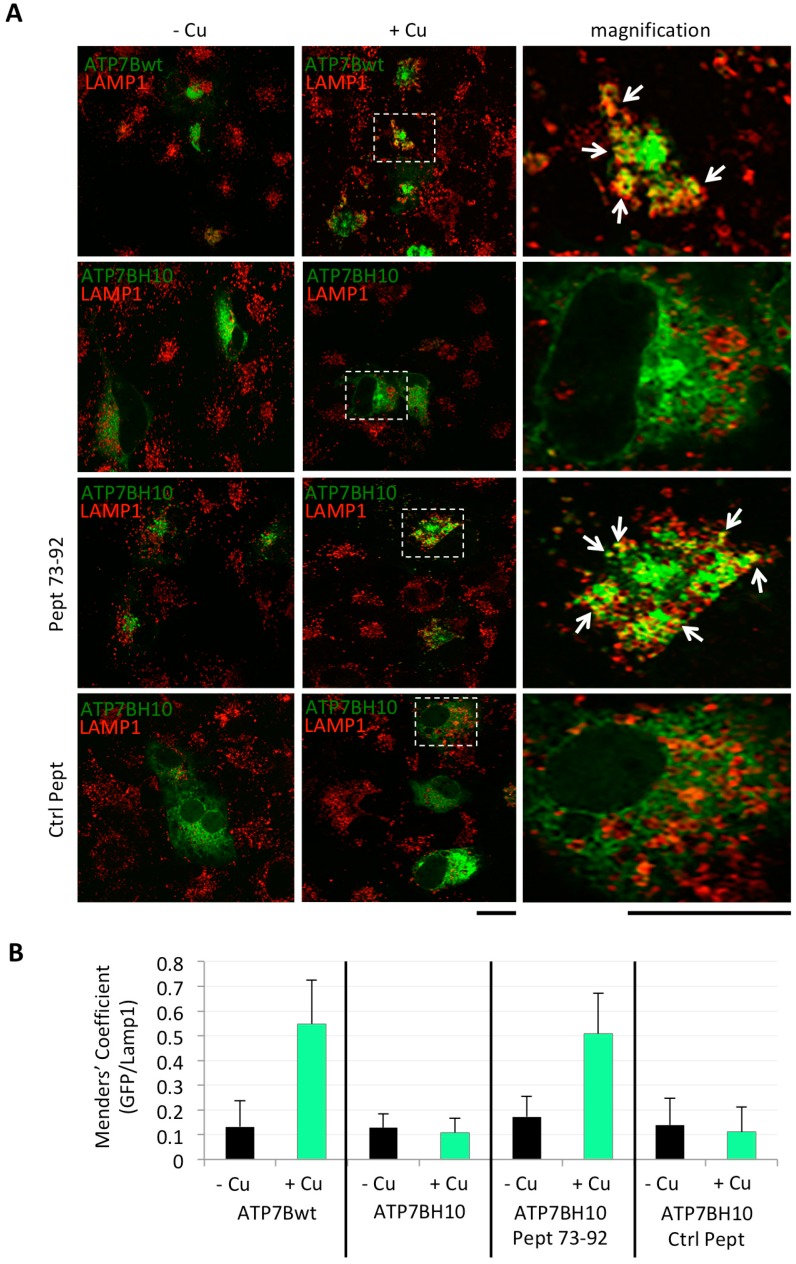
ATP7B-H1069Q, localized in the Golgi complex in cells incubated with Pept 73–92, moves to post-Golgi vesicles in response to Cu overload. (**A**) immunofluorescence analysis of COS7 cells transfected to express GFP-tagged ATP7B or ATP7B-H1069Q (upper two rows), incubated in the presence of untagged Pept 73–92 or control peptide 2 (Ctrl Pept) (lower two rows), and finally incubated in the presence of 200 μM CuSO_4_ as indicated on the top. After fixation, the cells were processed for double confocal immunofluorescence analysis to reveal the endo-lysosomal compartment with the marker LAMP-1. White arrows point to region of GFP and LAMP-1 overlapping signal. Scale bar 10 μm; (**B**) colocalization analysis of GFP and LAMP-1 signals (means ± SD, *n* = 20 cells from two independent experiments). Abbreviations as in Figure 1.

Encouraged by these findings, we next investigated whether the Golgi-corrected ATP7B-H1069Q was also able to be redistributed to the endolysosomal compartment upon Cu overload as ATP7B [14]. To address this question, COS7 cells were transfected to express GFP-tagged ATP7B or ATP7B-H1069Q, incubated as above with Pept 73–92 or control peptides, and finally incubated overnight in a medium containing 200 μM CuSO_4_. Strikingly, the confocal immunofluorescence analysis revealed that ATP7B, and Golgi-corrected ATP7B-H1069Q by the Pept 73–92, were able to move to post-Golgi vesicles visualised with the LAMP-1 lysosomal marker (Figure 3A). In contrast, in all other instances of Cu overload, as well as in the absence of Cu overload, almost no co-labelling of ATP7B forms and LAMP-1 was detected (Figure 3A).

This crucial result was quantified calculating the percentage of GFP signal of the ATP7B forms co-localizing with the LAMP-1 positive signal (Figure 3B). This methodological approach provided striking evidences of the specific and efficient effect exerted by the Pept 72–92 on the intracellular trafficking of ATP7B-H1069Q toward the endo-lysosomal compartment.

### 2.5. Pept 73–92 Interacts with ATP7B-H1069Q

**Figure 4:**
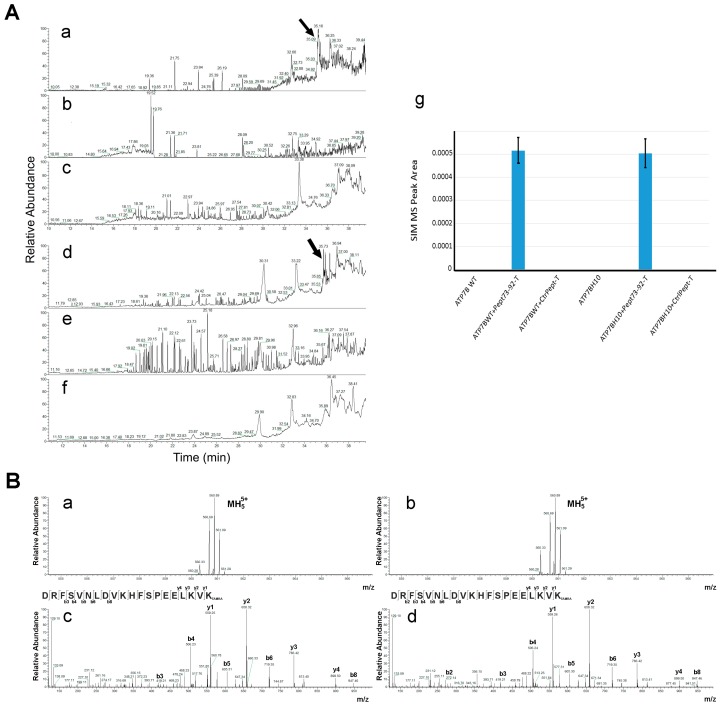
Pept 73–92 co-immunoprecipitates with ATP7B forms. (**A**) COS7 cells were transfected to express GFP-tagged ATP7B (subpanels a–c) or GFP-tagged ATP7B-H1069Q (subpanels d–f), incubated in the presence of TAMRA-tagged Pept 73–92 (subpanels a, d) or TAMRA-tagged control peptide 2 (subpanels b,e), lysed and immunoprecipitated with an anti-GFP antibody. The immunoprecipitated products were then analyzed by nanoLC-ESI-Q-Orbitrap SIM-MS under an experimental condition selectively monitoring the ion at *m*/*z* 560.69 ± 0.6 (see Material and Methods for details). Normalized area values of signals measured in subpanels a–f are reported in subpanel g; (**B**) mass spectrum of TAMRA-tagged Pept 73–92. Subpanels a and b: precursor quintuple charged ions at *m*/*z* 560.69 from panels Aa and Ad, respectively; subpanels c and d: corresponding MS/MS spectrum.

In order to elucidate the molecular mechanism by which Pept 73–92 was able to rescue the trafficking to the Golgi complex and, in the presence of Cu overload, the subsequent movement to the endo-lysosomal compartment of ATP7B-H1069Q, we asked whether the peptide physically interacts with the mutant protein. To this end, we first repeated the experimental plan shown in Figure 3, but 48 h post-transfection the cells were lysed and the GFP-tagged ATP7B forms were immuno-precipitated with anti-GFP antibody. The immuno-precipitated products were then subjected to mass spectrometric analysis. Pept 73–92 and control peptide tagged with TAMRA have the same molecular mass (2798.41 Da as monoisotopic value) and the same mass spectra. However, because of their different amino acid sequence, it is possible to distinguish them from their different fragmentation spectra. Since both of their mass spectra showed a prominent peak at *m*/*z* 560.69, which corresponds to the quintuple charged ion, we directly monitored for the presence of this signal in the immuno-precipitated material using the selected ion monitoring (SIM) mode to increase sensitivity of analysis. The SIM chromatograms shown in Figure 4A clearly show the presence of such product, eluting at about 35 min, in cells expressing ATP7B or ATP7B-H1069Q and incubated with TAMRA-tagged Pept 73–92 (panels a and d). Conversely, no signals were observed in the case of cells incubated with TAMRA-tagged control peptide or in cells incubated in the absence of peptides (panels b, c, f and g).

**Figure 5:**
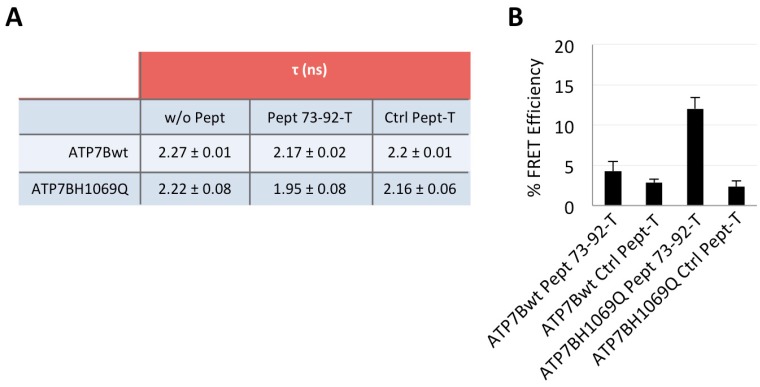
FLIM-FRET analysis of the interaction between TAMRA-tagged Pept 73–92 and ATP7B-H1069Q. COS7 cells were transfected to express GFP-tagged ATP7B or ATP7B-H1069Q, incubated in the presence of TAMRA-tagged Pept 73–92 or control peptide 1 as in Figure 2, fixed and analyzed. (**A**) lifetime values τ of ATP7B and ATP7B-H1069Q in the presence or in the absence of the peptides; (**B**) percentage of FRET efficiency (percentage of ATP7B and ATP7B-H1069Q τ decrease in the presence of peptides). Means ± SD, *n* = 10 cells from 2 independent experiments.

Next, we performed a Fluorescence Lifetime Imaging Microscopy (FLIM) analysis based on Fluorescence Resonance Energy Transfer (FRET) on cells manipulated as above and processed for confocal immunofluorescence microscopy [27,28]. FRET involves the transfer of energy from a fluorescent donor in its excited state to another excitable fluorophore (GFP and TAMRA, respectively, in our case). Indeed, FRET occurs when the distance between the donor and acceptor molecules is small (1–10 nm) and the fluorescence emission spectrum of the donor molecule overlaps to some extent the excitation spectrum of the acceptor, leading to a decrease of the donor fluorescence and reduction of excited state lifetime accompanied also by an increase in acceptor fluorescence intensity. FLIM measures the donor fluorescence lifetime τ which is reduced when FRET occurs for the energy transfer to the acceptor molecule, thus proving the interaction between two molecules. As shown in Figure 5A and Figure S4, we found that, only in cells expressing ATP7B-H1069Q and incubated with Pept 73–92, the τ value of the donor fluorescence lifetime decreased meaningfully (from 2.22 to 1.95 ns). This result was clearly emphasized when expressed as a FRET efficiency value (which represents the percent of lifetime decrease between a donor combined to an acceptor versus the donor alone, as reported in Figure 5B). It was 12%, indeed a quite significant value, whereas a minor value was obtained for ATP7B with Pept 73–92, only slightly above the background values measured with both constructs in the presence of the control peptide.

These results were further confirmed by the observation that cells not exhibiting the rescued Golgi localization of ATP7B-H1069Q failed to show a significant decrease of τ. In conclusion, both methods indicated a specific and most likely direct interaction of Pept 73–92 with ATP7B-H1069Q. Interestingly, the mass spectroscopic approach suggested a possible interaction of this peptide with ATP7B, an observation only partially supported by the FLIM-FRET procedure. This difference might be due to the extreme sensitivity of mass spectroscopy, or it may reveal an additional indirect interaction with ATP7B, different from the direct one occurring with the mutant protein and well detected by the FLIM-FRET procedure. Further work will be needed to clarify this perhaps only apparent discrepancy.

## 3. Discussion

The main finding reported here is the striking ability of Pept 73–92 peptide of the α-Crystallin domain of CRYAB protein to rescue in the living cells the proper localization and the subsequent trafficking in response to Cu overload of the WD-associated ATP7B-H1069Q mutant copper transporter. This result is reinforced by the observation that, differently from the N-terminal or C-terminal domains, also the isolated α-Crystallin domain, when expressed in the cell by transfection, maintains the rescue property exhibited by the full-length protein.

These data largely extend our previous conclusion on the specific effect of CRYAB on the folding and trafficking of ATP7B-H1069Q [10], as well as the evidence obtained by others on the properties of CRYAB Pept 73–92, namely: chaperone-like activity (anti-aggregation in vitro) [21]; anti-apoptotic effect in human lens epithelial cells, in mouse organ-cultured lenses and in human fetal RPE cells [22,23]; therapeutic effects in experimental autoimmune encephalomyelitis in mice and in cataract development in selenite-treated rats [22,24]. Although in these instances both targets and mechanisms of action of the peptide have yet to be determined, these results strongly support the outcome of our main finding: to deploy the Pept 73–92 for developing new effective drugs to contrast the form of Wilson disease caused by the ATP7B-H1069Q mutation, which is harbored in more than 50% of the Caucasian patients [6], firstly because the peptide shows clearly good access inside the cell, either in tissue culture or in vivo. In one instance, for human fetal RPE cells, its mechanism of entry has been characterized, showing the involvement of the sodium-coupled oligopeptide transporters 1 and 2 [23]. Most importantly, upon intraperitoneal injection, Pept 73–92 resulted in not only being able to reach the blood but also to cross the blood aqueous barrier to reach the lens in rats [22,24]. It is worth noting that such surprising findings have been confirmed using different peptides equivalent to CRYAB Pept 73–92 but derived from HspB1 and HspB6 proteins [29]. These results make reasonable the hypothesis that the Pept 73–92 might reach the liver from the blood and get access in the hepatocytes, which represent the main site responsible for the Cu homeostasis in our body. Moreover, in all instances reported in the literature, as well as in the present work, Pept 73–92 resulted in being effective when used in the micromolar range of concentration, an appropriate value for an effective drug.

On the basis of these considerations, and of the notion that ATP7B-H1069Q maintains a significant level of transport activity [9,15], unfortunately useless to excrete Cu from the cell due to its intracellular mis-localization, we plan in future work to test if Pept 72-93 is able to ameliorate/prevent the toxic accumulation of Cu in the liver of injected mice homozygotes for the H1069Q mutation. This is also because this form of Wilson disease is late onset and, despite significant progress in developing new cellular models [30,31], no cellular system in vitro can fairly reproduce the slow events occurring in vivo leading to the pathology. Mouse lines KO for ATP7B have been studied and show progressive accumulation of Cu in the liver and liver pathology in adults animals [32,33,34], thus mimicking the main landmark of Wilson disease in human. A mouse line apt for our study, *ATP7B^H1069Q/H1069Q^*, has been just obtained and made available for us (from Applied StemCell, City, CA, USA.; [35]), and is currently under characterization. This line should also allow for properly comparing the ability of Pept 73–92 with other previously proposed interventions for rescuing Cu-secretion disorders induced by ATP7B-H1069Q mutation, such as inhibitors of p38 and c-Jun N-terminal kinase signaling pathways [11,31].

Intriguingly, we demonstrate that Pept 73–92 and ATP7B-H1069Q protein most likely interacts directly. To the best of our knowledge, this would be the first identification of a molecular target for the peptide in the live cell. This result supports the hypothesis that the peptide acts as an amphipatic surface that binds unfolded/misfolded region of proteins, thus preventing their aggregation [24]. In this way, the peptide could also counteract the increased degradation rate of ATP7B-H1069Q, which is best made evident in hepatocytes-like cells [11,31]. Our data may also suggest that the peptide remains bound to the mutant transporter that reaches the Golgi complex. It would be very interesting in the future to understand if the peptide binds to single or multiple regions of the partially misfolded ATP7B-H1069Q protein. This is clearly a difficult task considering the size and the architecture of this membrane protein bearing seven transmembrane domains, but it should shed much light on understanding the folding of ATP7B. In addition, it is tempting to speculate how in the future structure–activity relation studies and drug design approaches might eventually lead to the identification of novel peptidomimetic molecules to be used as “functional agonist” of the ATP7B-H1069Q mutant for therapeutic purposes in Wilson disease.

## 4. Materials and Methods

### 4.1. cDNA Cloning and Plasmid Construction

Generation of ATP7B and full-length CRYAB was described elsewhere [10]. To generate HA-tagged CRYAB domains, N-terminal, α-crystallin, and C-terminal domains were amplified using the Expand High Fidelity PCR System (Merck, Darmstadt, Germany) and the following primers:

N-terminal domain
FW: 5′-GAATTCATGGACATCGCCATCCACC-3′
REV: 5′-CTCGAGCTATGAGAGAGTCCAGTGTCAAACC-3′
α-crystallin domain
FW: 5′-GAATTCCTCTCAGAGATGCGCCTGG-3′
REV: 5′-CTCGAGCTAATTCACAGTGAGGACCC-3′
C-terminal domain
FW: 5′-GAATTCCCAAGGAAACAGGTCTCTG-3′
REV: 5′-CTCGAGCTATTTCTTGGGGGCTGC-3’

The forward primers contain an EcoRI recognition site, whereas the reverse primers contain a XhoI recognition site. In the reverse primers used for the amplification of N-terminal and α-crystallin domains, a stop codon is also present. EcoRI and XhoI recognition sites are compatible with the multiple cloning sites on the HAN(I)-pcDNA3 expression vector. The amplified gene was cloned into pCR™2.1-TOPO-TA cloning vector (Invitrogen, Waltham, MA, USA) and transformed into TOP10 *Escherichia coli* cells. Transformed cells were selected on LB agar plates containing 100 µg/mL ampicillin at 37 °C for 16–18 h. Positive transformants were inoculated into LB broth containing 100 µg/mL ampicillin for plasmid propagation. Plasmid was isolated and the presence of the DNA fragment of interest was determined by restriction enzyme digestion and DNA sequencing. The TOPO vector containing the DNA fragment of interest and the expression vector were then digested with the same enzymes (EcoRI and XhoI) to generate the same sticky ends. The digestion products of both the DNA of interest and the expression vector were ligated at room temperature for 5 min using the Rapid DNA Ligation Kit (Roche, Diagnostics GmbH, Mannheim, Germany). Ligated mixture was transformed into TOP10 *E. coli* cells selected with the same strategies as those described above.

### 4.2. Cell Culture, Transfection, MTT Assay, Cell Fractionation and Immunoprecipitation

COS7 cells were routinely grown at 37 °C in Dulbecco’s modified essential medium (DMEM), containing 10% fetal bovine serum and transfected with X-tremeGene HP (Roche, Milan, Italy), according to the manufacturer’s instructions, using a 2:1 X-treme:DNA ratio. For MTT assay (Molecular probe Life Technologies, Eugene, OR, USA), cells were seeded in 96-well plates 10 μL of 5 mg/mL MTT solution was added to each well for 4 h at 37 °C. After removal of the medium, 100 μL of 0.1 M HCl in Isopropanol was added to each well to dissolve formazan crystals. The absorbance at 570 nm was determined using the SYNERGY H1 microplate reader (Bio-Tek Instruments, Inc, Winooski, VT, USA). For fractionation, cells were resuspended in lysis buffer (10 mM Tris-HCl pH 7.4, 150 mM NaCl, 1 mM EDTA pH 8.0, and protease inhibitors), lysed by sonication (Misonix XL-2000, New York, NY, USA) with 2 pulses of 3 s; intensity: 2. The samples were then centrifuged at 16,000× *g* at 4 °C for 30 min, the pellets resuspended in lysis buffer and the supernatants centrifuged at 100,000× *g* at 4 °C for 30 min. Immunoprecipitation was performed as detailed previously [10].

### 4.3. Peptides

The twenty-mer peptide DRFSVNLDVKHFSPEELKVK, corresponding to residue 73–92 of CRYAB, the inverted sequence peptide KVKLEEPSFHKVDLNVSFRD (control peptide 1), the scrambled peptide DLPLKVNVEDKFHRSFVESK (control peptide 2, and their 5-TAMRA tagged versions were purchased from GL Biochem (Shanghai, China) (see Figure S1 for details). Peptides were resuspended in H_2_O to obtain a final concentration of 250 μM each. In all the experiments, peptides were used at a final concentration of 12.5 μM. Both control peptides were used throughout the work and no difference in the results was detected.

### 4.4. Immunofluorescence

Confocal immunofluorescence analysis was performed as detailed previously [36] using a Zeiss LSM 510 Meta microscope (Carl Zeiss, Advanced Imaging Microscopy, Jena, Germany). The following antibodies were used: mouse monoclonal anti-HA and rabbit anti-GOLGA 2 (Sigma Aldrich, St. Louis, MO, USA); mouse monoclonal anti-LAMP-1 (DSHB, Iowa City, IA, USA).

### 4.5. Mass Spectrometry Analysis

Proteins/peptides were extracted from the immunoprecipitation beads with an aqueous 5% formic acid solution. The supernatants were transferred onto Centricon devices (3 kDa cutoff) (Merck Millipore, Darmstadt, Germany) and filtered by centrifugation at 14,000× *g* for 30 min. Resulting samples were dried and vacuumed, and then analyzed with a nanoLC-ESI-Q-Orbitrap MS/MS system, consisting of an UltiMate 3000 HPLC RSLC nano system-Dionex coupled to a Q-Exactive^Plus^ mass spectrometer through a Nanoflex ion source (Thermo Fisher Scientific, Waltham, MA, USA). Peptides were loaded on an Acclaim PepMap™ RSLC C18 column (150 mm × 75 μm ID, 2 μm—particles, 100 Å—pore size) (Thermo Fisher Scientific), and eluted with a gradient of solvent B (19.92/80/0.08 *v*/*v*/*v* water/acetonitrile/formic acid) in solvent A (99.9/0.1 *v*/*v* water/formic acid) at a flow rate of 300 nL/min. The gradient of solvent B started at 3%, increased to 10% over 5 min, increased to 30% over 40 min, rose to 80% over 5 min, remained at this percentage for 14 min, and finally returned to 3% in 1 min, remaining so for 30 additional minutes. The mass spectrometer operated in Selected Ion Monitoring (SIM) mode setting *m*/*z* = 560.69 ± 0.6 (charge 5+) with nominal resolution of 70,000, automatic gain control target of 50,000, and a maximum ion target of 100 ms, followed by MS/MS scans of the most abundant ion. MS/MS spectra were acquired in a scan *m*/*z* range 120–2000 using a normalized collision energy of 32%, an automatic gain control target of 200,000, a maximum ion target of 100 ms, and a resolution of 17,500. Two biological replicates were analyzed for each sample.

### 4.6. FLIM-FRET Analysis

FLIM-FRET analysis was performed 48 h post transfection on cells fixed with paraformaldehyde (2%) for 20 min. A TCS SMD SP5 Leica microscope (Leica Microsystems CMS, Am Friedensplatz, Mannheim, Germany) equipped with a FLIM module was used.

### 4.7. Statistical Analysis

The number of biological replicates of each experiment is indicated in the figure leg-ends. The means of at least 2 independent experiments were used to calculate SD. Significance of differences has been evaluated through Student’s *t*-test. Differences are only mentioned and interpreted as such if *p* < 0.005.

## Supplementary Materials

Supplementary materials can be found at http://www.mdpi.com/1422-0067/19/7/1892/s1.

## Abbreviations

WD	Wilson Disease
TGN	Trans-Golgi Network
ER	Endoplasmic Reticulum
CRYAB	αB-Crystallin
GFP	Green Fluorescent Protein
HA	Hemagglutinin
ACD	α-Crystallin Domain
NTD	N-Terminal Domain
CTD	C-Terminal Domain
TAMRA	Tetramethylrhodamine Azide
MTT	3-(4,5-Dimethylthiazol-2-yl)-2,5-Diphenyltetrazolium Bromide
FLIM	Fluorescence Lifetime Imaging Microscopy
FRET	Fluorescence Resonance Energy Transfer
