# Supplementary material for: An αB-Crystallin Peptide Rescues Compartmentalization and Trafficking Response to Cu Overload of ATP7B-H1069Q, the Most Frequent Cause of Wilson Disease in the Caucasian Population
Source: Int J Mol Sci. 2018 Jun 27;19(7):1892. doi: 10.3390/ijms19071892 (PMC6073935; doi:10.3390/ijms19071892)
Supplement: Supplementary file 1 [file ijms-19-01892-s001.pdf]

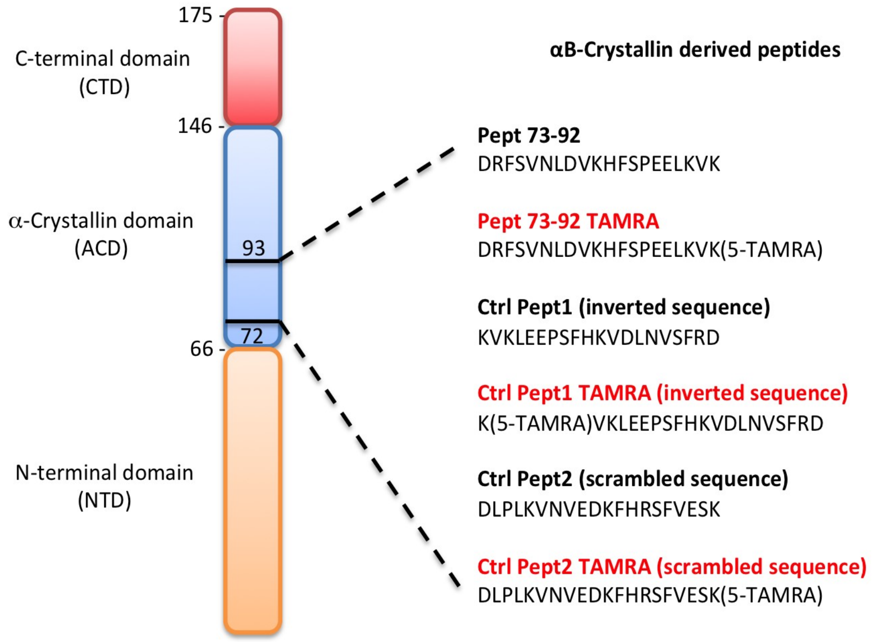

Fig. S1. Schematic drawing of αB-crystallin domains and of the peptides used in this work.

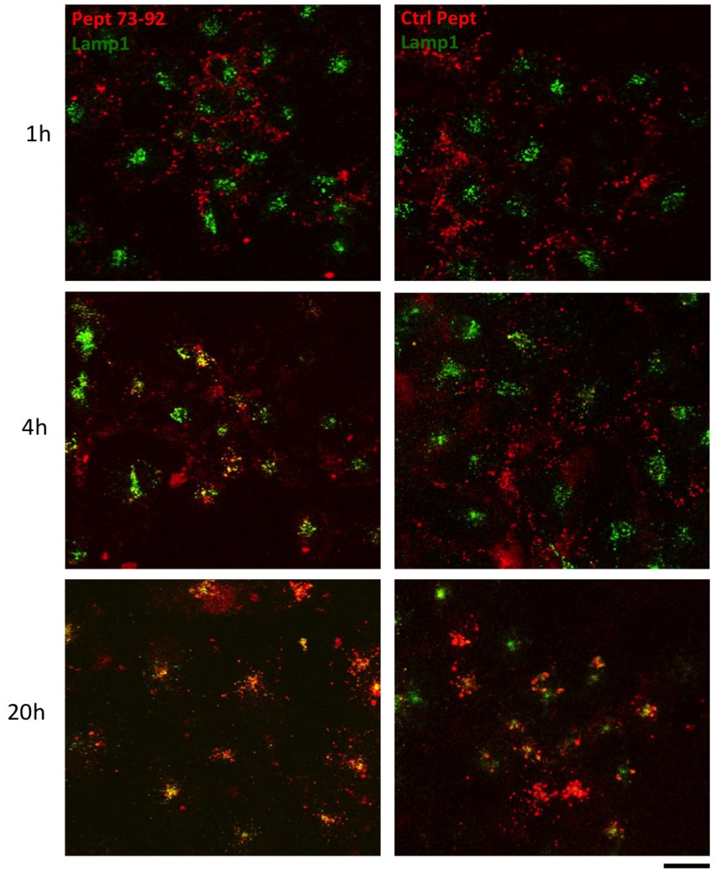

Fig. S2. Confocal immunofluorescence analysis of the uptake of TAMRA-tagged Pept 73-92 and control peptide 1. COS7 cells were incubated in the presence of the TAMRA version of Pept 73-92 (left panels) or control pept 1 (Ctrl Pept) (right panels) for the time indicated on the left. Cells were then fixed and processed to reveal the peptides and the endo-lysosomal compartment with the LAMP1 protein marker as indicated. Scale bar 10 µm.

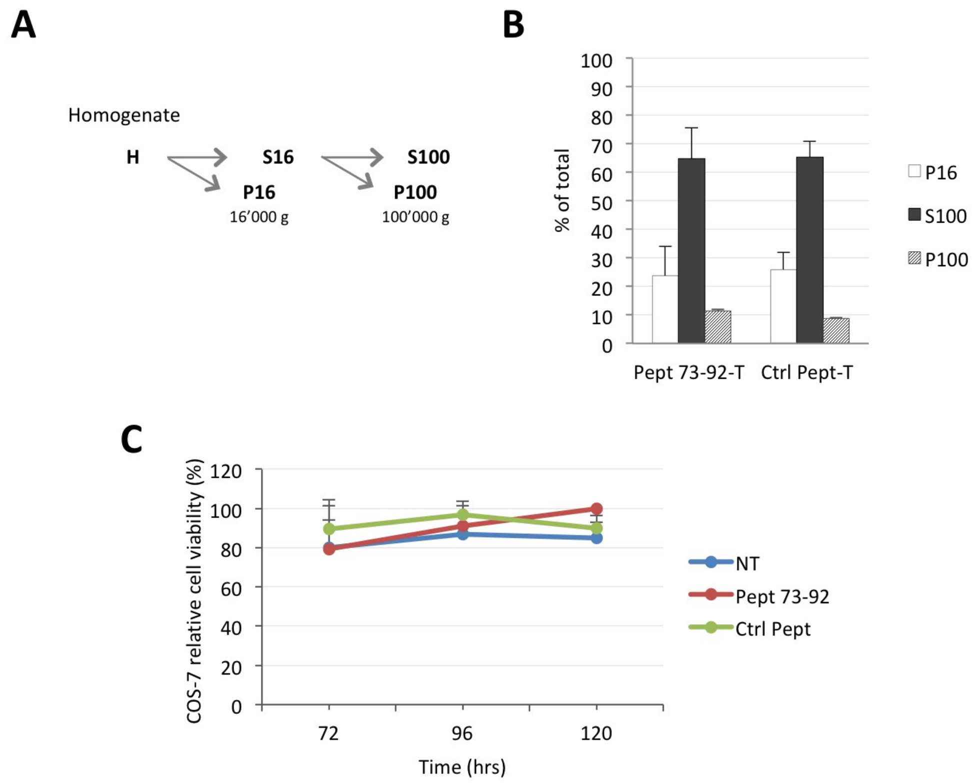

Fig. S3. Intracellular distribution of TAMRA-tagged Pept 73-92 and control peptide 1, and corresponding viability of the cells. A) Schematic drawing of the cell fractionation of COS7 cells incubated for 24 h in the presence of TAMRA-tagged Pept 73-92 (Pept 73-92-T) or control pept 1 (Ctrl Pept-T). B) Relative distribution of the peptides within the indicated cellular fractions. C) Cell viability of COS7 cells incubated in the absence or in the presence of TAMRA-tagged Pept 73-92 (Pept 73-92) or control pept 1 (Ctrl Pept) for the indicated time. Means +/- SD from two independent experiments.

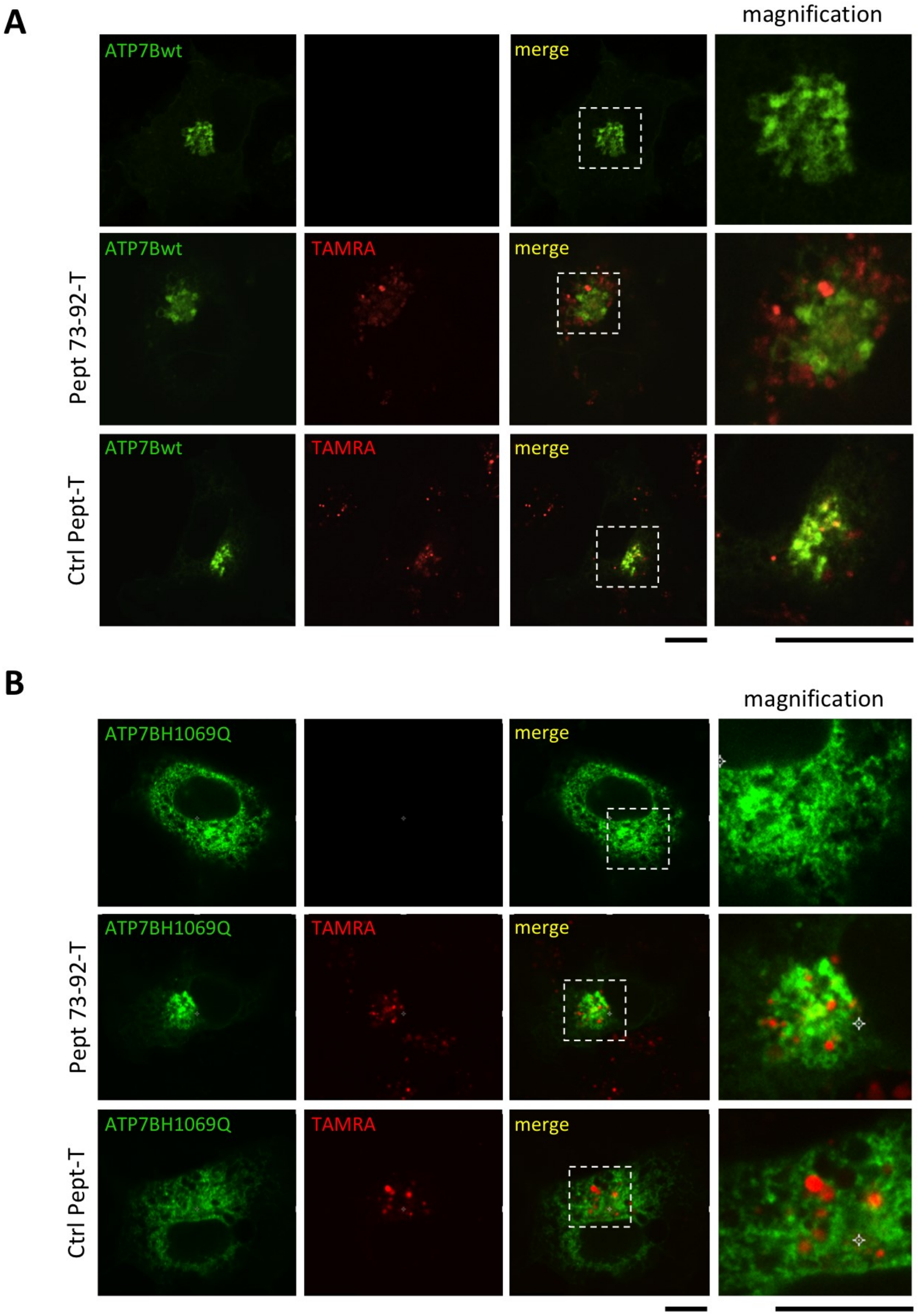

Fig. S4. Sample of the images used for the FLIM-FRET analysis (see Fig. 5). A) and B) ATP7B and ATP7B-H1069Q transfected cells, respectively, incubated in the absence or in the presence of TAMRA-tagged Pept 73-92 (Pept 73-92-T) or control pept 1 (Ctrl Pept-T). Scale bars 10 µm.
